# The Contribution of Avoidable Mortality to the Life Expectancy Gains in Korea between 1998 and 2017

**DOI:** 10.3390/ijerph17186499

**Published:** 2020-09-07

**Authors:** Jinwook Bahk, Kyunghee Jung-Choi

**Affiliations:** 1 Department of Public Health, Keimyung University, 1095, Dalgubeol-daero, Dalseo-gu, Daegu 42601, Korea; jwbahk@kmu.ac.kr; 2 Department of Occupational and Environmental Medicine, College of Medicine, Ewha Womans University, 25, Magokdong-ro 2-gil, Gangseo-gu, Seoul 07804, Korea

**Keywords:** life expectancy, avoidable mortality, trends, decomposition, Korea

## Abstract

This study evaluated the contribution of avoidable causes of death to gains in life expectancy between 1998 and 2017 in Korea. This is a multi-year, cross-sectional study using national data. Death certificate data from 1998 to 2017 were obtained from Statistics Korea. The difference in life expectancy between 1998 and 2017 by age and cause of death were decomposed using Arriaga’s method. Life expectancy rose 7.73 years over 20 years in Korea, which was largely (more than 50%) due to changes in avoidable causes of death. As age increased, the contribution to changes in life expectancy increased, and the gain in life expectancy due to avoidable causes also tended to increase. The major factors that drove that gain in life expectancy were avoidable causes such as cerebrovascular diseases and traffic accidents. The gain in life expectancy from preventable diseases was greater in men than in women. The results of this study indicate that active public health programs have been effective in improving life expectancy in Korea. Moreover, avoidable mortality could be further improved with good public health policy. Health policy aimed at reducing amenable and preventable deaths should be further implemented to promote population health.

## 1. Introduction

South Korea (hereafter Korea) is one of the countries predicted to have the longest life expectancy by 2030 [1]. In the past 50 years, life expectancy at birth in Korea has increased at a higher rate than other Organization for Economic Co-operation and Development (OECD) countries [2]. This rapid increase in life expectancy was attributed to socioeconomic development, subsequent improvements in living standards, substantial lifestyle changes due to urbanization and industrialization, and improved healthcare [3]. In addition, changes in life expectancy can be affected by major social changes, such as a financial crisis, which can influence public health budgets [4,5].

Avoidable mortality is defined as mortality that can be saved via timely interventions and effective public health [2]. Avoidable mortality was proposed by Rutstein et al. in 1976 [6], but the precise definition of avoidable death is still controversial [7,8,9,10]. However, avoidable mortality can be useful as an indicator of health system performance. The OECD has been monitoring avoidable mortality—preventable mortality and treatable mortality—in OECD countries [11]. Most OECD countries have reported a declining trend of avoidable mortality since 2000. Among OECD countries, Korea had relatively low levels of preventable mortality and treatable mortality in 2017 [2].

The contribution of avoidable mortality in life expectancy changes should be evaluated to assess the performance of the public health system. In Argentina, Chile, Colombia, and Mexico, non-avoidable mortality contributed to life expectancy gains greater than avoidable mortality between 2000–2011 [12]. This was true in Spain from 1987 to 2001 [13]. The increase in avoidable deaths contributed to shorted life expectancy in Lithuania during 2001–2008 [14]. However, limited studies have explored the contribution of the public health system in Korea, where life expectancy has steadily and steeply risen, on life expectancy. We hypothesize that avoidable mortality has contributed to the increase in life expectancy in Korea over the past 20 years. The aim of this study is to evaluate the contribution of avoidable death on changes in life expectancy between 1998 and 2017 in Korea. We analyzed the contribution of avoidable mortality by gender, age, and causes of death for 20 years.

## 2. Materials and Methods

### 2.1. Study Design Data Sources, and Study Size

This is a multi-year, cross-sectional analysis on life expectancy contributions using national population and mortality data. This work follows the Strengthening the Reporting of Observational Studies in Epidemiology (STROBE) guidelines for cross-sectional studies [15]. The numbers of deaths and population according to the calendar years (1998 to 2017), gender, age (0, 1–4, 5–9, 10–11, …, 85+) were obtained from death certificate data and resident registration data, respectively, provided by Statistics Korea. Mortality data (death certificate data) from 1998 to 2017 were obtained from Microdata Integrated Service (http://mdis.kostat.go.kr/). Mortality data included the causes of death as defined by the International Classification of Diseases (ICD) revision 10. Mid-year estimates of resident population were used as population denominators. Mid-year populations from 1998 to 2017 were obtained from the Korean Statistical Information Service (http://kosis.kr/). Mortality data and population data cover the entire registered Korean population except for foreigners. A total of 985,047,593 subjects and 5,112,119 deaths (including 771,704 amenable deaths and 1,606,119 preventable deaths) were analyzed from 1998 to 2017 (see Supplementary Table S1).

### 2.2. Variables

Population and mortality data were categorized into 5-year age groups (0, 1–4, 5–9, 10–14, …, 80–84, 85+) and subdivided according to sex and year. Avoidable deaths were subdivided into amenable and preventable deaths. This categorization was conducted based on the proposal by the UK Office for National Statistics (ONS) in 2011 [7]. The list of amenable causes defined by Nolte and McKee [16] is widely used. However, since the current study considered both amenable and preventable deaths, the list provided by the UK ONS was more appropriate because it provides both lists simultaneously. The list of causes of death considered amenable and preventable was presented in Supplementary Table S2.

### 2.3. Statistical Analysis

Annual period life tables from 1998 to 2017 were constructed using 5-year probabilities of death by applying standard life table procedures [17]. Annual life expectancies were presented in Supplementary Table S3. Life expectancy differences between 1998 and 2017 by age and cause of death were decomposed using Arriaga’s method [18]. Decomposition analyses were conducted for all years from 1999 to 2017 with 1998 acting as a baseline (e.g., between 1998 and 1999, between 1998 and 2000, …, between 1998 and 2017). The difference in life expectancy between two years was considered to be the sum of the age- and cause-specific components. The difference in life expectancy was decomposed according to the contribution of each age group. Following this decomposition, age-specific contributions were separated according to the contributions of each specific cause of death. A more detailed description and analysis of Arriaga’s decomposition method is described elsewhere [17,18,19]. SAS code for decomposing life expectancy differences between years by age and cause of death using Arriaga’s decomposition method has been previously published in Auger et al. [19].

### 2.4. Research Ethics

The study was approved by the Keimyung University Institutional Review Board (IRB No. 40525-201712-HR-84-01). The study only used publicly available secondary data without any personal identifiers.

## 3. Results

### 3.1. Contribution to the Life Expectancy Gain between 1998 and 2017

**Figure 1:**
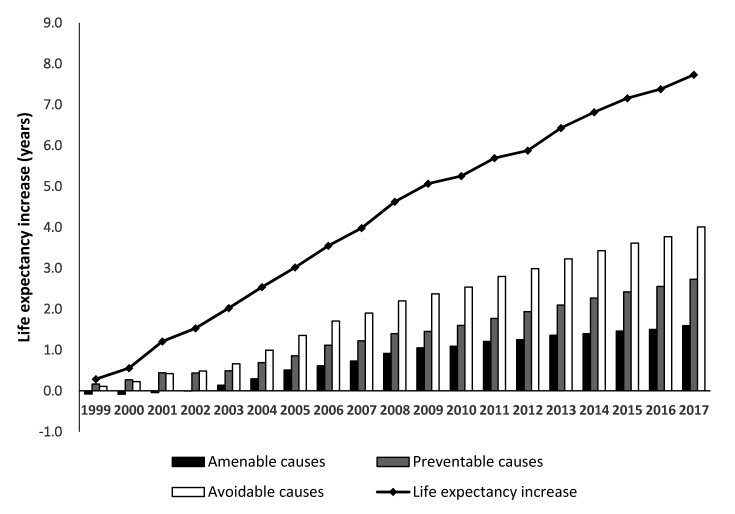
Contributions of amenable, preventable, and avoidable causes to cumulative increases in life expectancy based on life expectancy in 1998 in Korea (men and women combined).

Figure 1 shows the cumulative contributions of amenable, preventable, and avoidable causes of death on increased life expectancy between 1998 and 2017 in Korea. Over the last two decades, life expectancy steadily increased by 7.73 years. A reduction in preventable deaths underlay an increase of 2.725 years of life expectancy, while amenable deaths contributed 1.597 years. An overall reduction in avoidable deaths, which includes both amenable and preventable deaths, contributed 51.9% (4.010 years) to the increase in life expectancy (see Supplementary Tables S4 and S5).

**Figure 2:**
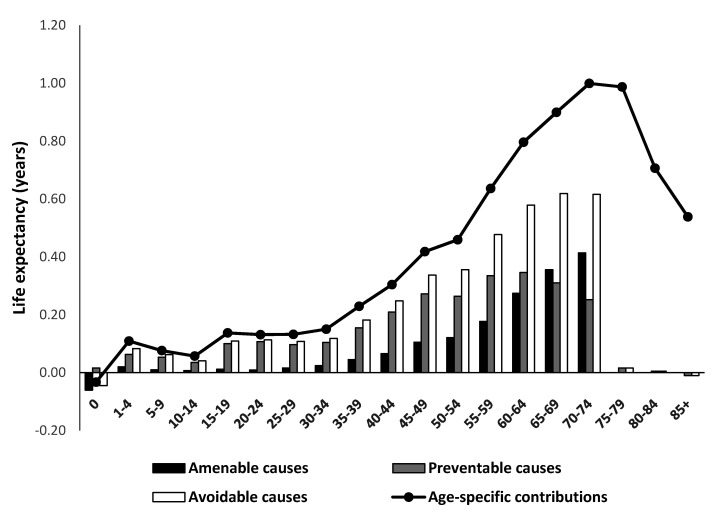
Age-specific contributions of amenable, preventable, and avoidable causes to life expectancy differences between 1998 and 2017 in Korea (men and women combined).

Figure 2 presents the age-specific contributions of amenable and preventable deaths to the life expectancy gain between 1998 and 2017. The older age group of more than 60 years old significantly contributed to the increase in life expectancy, which accounts for 4.9 years (63.7%) of the total increase (see Supplementary Table S6 for more detailed values). As age increased, the contribution to changes in life expectancy increased, and the gain in life expectancy due to avoidable causes also tended to increase. For example, in the 70–74-year-old age group, a reduction in amenable causes of death led to an increase of 0.414 years (41.1%) in life expectancy. Additionally, preventable causes added 0.346 years to life expectancy for the 60–64-year-old group. For amenable causes of death, the 55–74-year-old group accounted for 76.6% of the gain in life expectancy. Subjects aged 45–74 years old contributed 56.0% of the life expectancy increases due to preventable causes of deaths (see Supplementary Table S6).

### 3.2. Cause-Specific Contributions

Table 1 shows cause-specific contributions of amenable and preventable deaths to the life expectancy differences between 1998 and 2017. The major factor that controlled the effect amenable causes had on life expectancy between 1998 and 2017 were reductions in cerebrovascular disease-related death in both men and women, which resulted in a gain of 0.999 years in life expectancy for men and 0.957 years for women. Transport accidents were the main contributor of preventable causes to life expectancy differences in both men and women. Reductions in transport accidents contributed to 0.806 years of gain in life expectancy for men and 0.339 years for women. The second highest contributor to preventable causes of death was alcohol-related disease in men and stomach cancer in women (see Supplementary Figure S1).

## 4. Discussion

This study found that differences in avoidable causes of death had a 50% effect on the increase of 7.73 years in life expectancy in Korea from 1998 to 2017. As age increased, the contribution to changes in life expectancy increased, and the gain in life expectancy due to avoidable causes also tended to increase. The major contributors to changes in life expectancy were cerebrovascular diseases and traffic accidents, both of which are avoidable causes. The life expectancy gain from preventable diseases was greater in men than in women.

In the past 20 years, life expectancy in Korea increased 7.73 years from 75.40 to 83.13. This change was largely affected by increased life expectancy in older people (more than 60 years old), as they accounted for 4.9 years (63.7%). This pattern is very different from previous increases in life expectancy in Korea. Infant mortality reduction largely drove the gain in life expectancy between 1970 and 2005 in Korea [3], whereas it did not contribute significantly between 1998 and 2020. Importantly, infant mortality rates, as well as other disease mortality rates, did decrease from 6.20 per 1000 live births in 1999 [20] to 2.80 per 1000 live births in 2017, which was less than average infant mortality rates (3.5 deaths per 1000 live births) in OECD countries [2]. However, the reduction in mortality in the older age group (65 years old or over), which decreased from 4845.9 per 100,000 in 1998 to 3080.9 per 100,000 in 2017, had a far greater impact than infant mortality [21]. This pattern follows the pattern of other countries, in that, initial increases in life expectancy were largely due to decreasing mortality rates in younger people, while later increases were more significantly affected by survival after age 65 [22,23].

Avoidable deaths, an indicator of the quality of medical care and prevention interventions [6], contributed to a 4.010 year (51.9%) increase in life expectancy over 20 years in Korea. Although direct comparison with other studies is difficult, due to different study periods and different lists of avoidable deaths, the contribution of 51.9% seems to be greater than in other countries [12,13]. The major factor that underlay the changes in amenable causes was cerebrovascular disease in both men and women, which contributed to 0.999 years gain in life expectancy for men and 0.957 years for women. Those accounted substantially for the 57.7% and 66.9% effect amenable causes had on life expectancy gain, respectively. In Europe, studies indicated that changes in circulatory system disease-related death were the main factor affecting life expectancy [24]. The age-adjusted death rate from cerebrovascular disease in Korea decreased from 96.4 per 100,000 in 1998 to 24.5 in 2017 [25]. Although all OECD countries showed a decreasing pattern in stroke-related mortality rates from 2000 to 2017, the reduction in stroke mortality in Korea was substantially higher (−66%) than the average of OECD countries (−47%) [2]. The reduction of stroke mortality might be attributable to efforts for health care quality improvements. To reduce mortality and increase survival rate, the acute stroke quality assessment program (ASQAP) was implemented in 2005 by the Health Insurance Review and Assessment Service (HIRA), the Ministry of Health and Welfare established the Regional Comprehensive Stroke Centers (CSC) from 2008 [26], and the Korean Stroke Society started to certify the stroke unit in 2012 [27]. These programs attributed to the improvement of quality care for acute stroke patients [28]. Mortality following ischemic stroke was 6.2 per 100 patients in 2017, which was about half of the OECD average of 12.3 [2]. Moreover, the treatment rate of hypertension, which is the main preventable factor for cerebrovascular disease, increased from 22% in 1998, 50% in 2005, to 61% in 2016 [29].

Among preventable causes, reductions in transport accidents were the greatest contributor to improved life expectancy in both men and women; specifically, transport accidents contributed to 0.806 years gained for men and 0.339 years for women. While mortality rates from transport accidents in Korea were still high among OECD countries in 2017, the reduction in transport accidents mortality from 1998 to 2017 was substantial [30,31]. This decreasing trend may reflect the nation’s prosperity [32]. The relationship between economic development and traffic accident mortality has been reported to be non-linear; economic development initially increases traffic-related deaths, but later prevented those deaths. This seemed to be because of improvements in transportation infrastructure and medical trauma care. Under the pressure of non-governmental organizations, the Korean government responded to road the traffic accidents issue with the following policy interventions beginning in the mid-1990s [33,34]: application of a road safety inspection system, road structure change for risky roads, expansion of traffic-monitoring cameras, more severe penalties for risky driving behaviors, financial rewards for citizens reporting traffic violations, and road safety education programs.

The life expectancy gain from preventable causes was greater in men than in women, mainly due to alcohol-related disease, accidental injury, liver cancer, lung cancer, and stomach cancer, as well as transport accidents. These death causes were the typical causes of death correlated with the higher mortality rates in men than in women. Alcohol consumption leads to alcohol-related disease and liver cancer. However, consumption of alcohol in Korea has not decreased over the last 20 years [2]. Rather, declining trends of hepatitis B virus infections from active immunization programs could affect the reduction of mortality from alcohol-related disease and liver cancer [35]. The Korean government introduced comprehensive anti-smoking measures in 1995 through the enactment of the Health Promotion Act and ratified the World Health Organization Framework Convention on Tobacco Control (WHO FCTC) in 2005 [36]. The smoking rate in Korean men decreased from 71.7% in 1992 to 39.7% in 2016, while female smoking rates remained very low [37,38]. Moreover, the seroprevalence of Helicobacter pylori infection in asymptomatic subjects, which is affected by the early-life social environment [39], decreased since 2008 [40]. In addition, in Korea, the five-year net survival from lung cancer, stomach cancer, colon cancer, and rectal cancer showed a considerable improvement that was higher than the OECD average [2].

As stated above, avoidable causes of death contributed greater absolutely to the gains in life expectancy in older population. Especially in the 65–74-year-old age groups, amenable causes contributed a life expectancy gain of 0.770 years, which was greater than the contribution from preventable causes (0.562 years). Korea was one of the OECD countries in which the gain of life expectancy at age 65 between 1970 and 2017 was the highest [2]. Although the poverty rate for people aged 65 and over was very high in OECD countries [41], Korea has universal coverage for health services through the national health system [2]. Furthermore, a long-term care system for the older population was introduced in 2008, and beds for long-term care were markedly increased from 2008 to 2017. The per-capita medical expenses of the older population have increased continuously since 2008 [42]. In addition, in Korea, 83% of the population aged 65 and over were covered for vaccination against influenza 2017, which is above the 75% target among OECD countries [2]. However, prevention programs, such as hepatitis B vaccination or smoking cessation, usually take time to fully affect death rates, and the effect can be maximized when the intervention took place at an early age.

This study has limitations. First, we used all deaths registered between 1998 and 2017. Korean death registration is regarded as almost completely updated for all deaths occurring among those aged 1+ years since the mid-1980s [43]. However, the information regarding causes of death in the death certificate data for infant deaths and the elderly population may be incomplete [44,45]. In these cases, the cause of death is registered as R code. This could lead to underestimating the magnitude of the deaths from amenable and preventable causes. Second, there are many versions of avoidable death classification, which could hinder comparisons with various research results from other countries.

In Korea, which offers universal coverage for health services through a national health system, active public health programs have been effective in raising life expectancy. However, there is room for improvement for avoidable mortality. Health policies that reduce amenable and preventable mortality should be further implemented. Furthermore, future studies are needed to evaluate the specific contribution of the public health system on avoidable death by using various analysis methods.

## 5. Conclusions

In the Korean population, life expectancy rose 7.73 years between 1998 and 2017. A reduction in avoidable death causes contributed to this gain by more than 50%. The reduction in mortality in the older age group contributed mainly to the gains in life expectancy, which was significantly affected by the reductions in avoidable causes of death. The major contributors to life expectancy gain were cerebrovascular diseases and traffic accidents. The life expectancy gain from preventable diseases was greater in men than in women. Active public health programs in Korea attributed to the rise in life expectancy over the last 20 years. However, there is room for improvement in avoidable mortality. Health policies to reduce amenable and preventable mortality should be further implemented.

## Figures and Tables

**Table 1:** Amenable and preventable cause-specific contributions to life expectancy differences between 1998 and 2017 in Korea.

Causes	Amenable			Preventable		
	All	Men	Women	All	Men	Women
**Infections**						
Tuberculosis	0.137	0.198	0.063	0.137	0.198	0.063
Selected invasive bacterial and protozoal infections	0.060	0.075	0.043			
Hepatitis C	−0.001	−0.001	−0.001	−0.001	−0.001	−0.001
HIV/AIDS	−0.002	−0.003	0.000	−0.002	−0.003	0.000
**Neoplasms**						
Malignant neoplasm of lip, oral cavity and pharynx				−0.001	0.001	0.001
Malignant neoplasm of esophagus				0.043	0.077	0.008
Malignant neoplasm of stomach				0.391	0.493	0.272
Malignant neoplasm of colon and rectum	0.011	−0.002	0.036	0.011	−0.002	0.036
Malignant neoplasm of liver				0.278	0.405	0.138
Malignant neoplasm of trachea, bronchus and lung				0.183	0.302	0.078
Malignant melanoma of skin	−0.001	0.000	−0.002	−0.001	0.000	−0.002
Mesothelioma				−0.002	−0.002	0.000
Malignant neoplasm of breast	−0.019	0.000	−0.048	−0.019	0.000	−0.048
Malignant neoplasm of cervix uteri	0.011	0.000	0.022	0.011	0.000	0.022
Malignant neoplasm of bladder	0.006	0.010	0.003			
Malignant neoplasm of thyroid gland	0.004	0.003	0.005			
Hodgkin’s disease	−0.001	−0.001	0.000			
Leukemia	0.002	0.001	0.003			
Benign neoplasms	0.001	0.001	0.001			
**Nutritional, endocrine and metabolic**						
Diabetes mellitus	0.032	0.045	0.015	0.032	0.045	0.015
**Drug use disorders**						
Alcohol related diseases, excluding external causes				0.442	0.699	0.127
Illicit drug use disorders				0.000	0.000	0.000
**Neurological disorders**						
Epilepsy and status epilepticus	0.013	0.014	0.011			
**Cardiovascular diseases**						
Rheumatic and other valvular heart disease	0.002	0.001	0.003			
Hypertensive diseases	0.103	0.095	0.108			
Ischemic heart disease	0.146	0.164	0.134	0.146	0.164	0.134
DVT with pulmonary embolism				−0.002	0.000	−0.003
Cerebrovascular diseases	0.992	0.999	0.957			
Aortic aneurysm and dissection				0.001	−0.001	0.004
**Respiratory diseases**						
Influenza (including swine flu)	0.001	0.001	0.001	0.001	0.001	0.001
Pneumonia	0.004	0.006	0.014			
Chronic obstructive pulmonary disorder				0.037	0.047	0.031
Asthma	0.078	0.095	0.056			
**Digestive disorders**						
Gastric and duodenal ulcer	0.017	0.024	0.008			
Acute abdomen, appendicitis, intestinal obstruction, cholecystitis/lithiasis, pancreatitis, hernia	0.018	0.024	0.011			
**Genitourinary disorders**						
Nephritis and nephrosis	0.052	0.057	0.048			
Obstructive uropathy and prostatic hyperplasia	0.001	0.001	0.000			
**Maternal and infant**						
Complications of perinatal period	−0.088	−0.095	−0.080			
Congenital malformations, deformations and chromosomal anomalies	0.018	0.017	0.019			
**Unintentional injuries**						
Transport Accidents				0.598	0.806	0.339
Accidental Injury				0.385	0.527	0.216
**Intentional injuries**						
Suicide and self-inflicted injuries				0.014	0.061	−0.021
Homicide/Assault				0.043	0.046	0.037
Misadventures to patients during surgical and medical care	0.000	0.000	0.001	0.000	0.000	0.001
**Total contribution (years)**	**1.60**	**1.73**	**1.43**	**2.73**	**3.86**	**1.45**

## Supplementary Materials

The following are available online at https://www.mdpi.com/1660-4601/17/18/6499/s1, Figure S1: The major amenable and preventable causes of death contributed to life expectancy difference between 1998 and 2017 by sex, Table S1: Annual numbers of mid-year population and deaths. Table S2: The list of causes of death considered amenable and preventable, Table S3: Life expectancy at birth and annual increases (years) from 1998 to 2017, Table S4: Cause-specific contributions to the life expectancy difference (years) between 1998 and 2017, Table S5: Age-specific contributions to the life expectancy difference (years) between 1998 and 2017 by sex, Table S6: Contributions to cumulative increase in life expectancy (years) based on the life expectancy at 1998 by sex.
