# Supplementary material for: The Contribution of Avoidable Mortality to the Life Expectancy Gains in Korea between 1998 and 2017
Source: Int J Environ Res Public Health. 2020 Sep 7;17(18):6499. doi: 10.3390/ijerph17186499 (PMC7558962; doi:10.3390/ijerph17186499)
Supplement: Supplementary file 1 [file ijerph-17-06499-s001.zip › Supplementary materials.docx]

Supplementary Table S1. Annual numbers of mid-year population and deaths

| 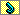 | No. of population | No. of deaths | No. of amenable deaths | No. of preventable deaths | No. of avoidable deaths |
| --- | --- | --- | --- | --- | --- |
| **Men and women combined** | |  |  |  |  |
| 1998 | 46837620 | 245825 | 43538 | 82309 | 113317 |
| 1999 | 47163425 | 247734 | 44767 | 81786 | 113062 |
| 2000 | 47534117 | 248740 | 46224 | 82507 | 114373 |
| 2001 | 47877049 | 243813 | 46157 | 81987 | 113614 |
| 2002 | 48125745 | 247524 | 46997 | 84973 | 116154 |
| 2003 | 48308386 | 246463 | 45305 | 86834 | 116187 |
| 2004 | 48485314 | 246220 | 43667 | 85750 | 113435 |
| 2005 | 48683040 | 245874 | 41130 | 84764 | 109911 |
| 2006 | 48887027 | 244162 | 40254 | 82326 | 106332 |
| 2007 | 49130354 | 246482 | 39276 | 82275 | 105399 |
| 2008 | 49404648 | 246113 | 37220 | 81044 | 103133 |
| 2009 | 49656756 | 246942 | 35387 | 82302 | 102499 |
| 2010 | 49879812 | 255405 | 35205 | 81442 | 101597 |
| 2011 | 50111476 | 257396 | 33658 | 79757 | 98738 |
| 2012 | 50345325 | 267221 | 33683 | 78395 | 97321 |
| 2013 | 50558952 | 266257 | 32648 | 77386 | 95682 |
| 2014 | 50763158 | 267692 | 32483 | 75288 | 93309 |
| 2015 | 50951719 | 275895 | 31982 | 73781 | 91739 |
| 2016 | 51112972 | 280827 | 31738 | 72068 | 89916 |
| 2017 | 51230704 | 285534 | 30385 | 69145 | 86157 |
| Total | 985047593 | 5112119 | 771704 | 1606119 | 2081875 |
| **Men** |  |  |  |  |  |
| 1998 | 23519754 | 137879 | 25618 | 60268 | 78273 |
| 1999 | 23679249 | 137637 | 26227 | 59494 | 77656 |
| 2000 | 23863276 | 137789 | 26908 | 59706 | 78087 |
| 2001 | 24032276 | 135218 | 27223 | 59066 | 77624 |
| 2002 | 24151328 | 135846 | 27286 | 60685 | 78678 |
| 2003 | 24235168 | 135885 | 26493 | 61845 | 79010 |
| 2004 | 24316613 | 136235 | 25595 | 61546 | 77790 |
| 2005 | 24409659 | 135317 | 24314 | 60132 | 75096 |
| 2006 | 24506619 | 134631 | 24145 | 58669 | 73235 |
| 2007 | 24624127 | 135664 | 23467 | 58084 | 72000 |
| 2008 | 24757073 | 136932 | 22528 | 57410 | 71007 |
| 2009 | 24876418 | 137735 | 21543 | 58133 | 70732 |
| 2010 | 24977164 | 142358 | 21509 | 57795 | 70442 |
| 2011 | 25081788 | 143250 | 20652 | 56821 | 68863 |
| 2012 | 25187494 | 147372 | 20916 | 55864 | 67974 |
| 2013 | 25282928 | 146599 | 20143 | 54873 | 66665 |
| 2014 | 25374486 | 147321 | 20108 | 53530 | 65206 |
| 2015 | 25458058 | 150449 | 20049 | 52316 | 64124 |
| 2016 | 25527815 | 152529 | 19934 | 51242 | 63046 |
| 2017 | 25576752 | 154328 | 19416 | 49689 | 61110 |
| Total | 493438039 | 2820974 | 464074 | 1147168 | 1436618 |
| **Women** |  |  |  |  |  |
| 1998 | 23317867 | 107946 | 17920 | 22041 | 35044 |
| 1999 | 23484176 | 110097 | 18540 | 22292 | 35406 |
| 2000 | 23670842 | 110951 | 19316 | 22801 | 36286 |
| 2001 | 23844773 | 108595 | 18934 | 22921 | 35990 |
| 2002 | 23974417 | 111678 | 19711 | 24288 | 37476 |
| 2003 | 24073218 | 110578 | 18812 | 24989 | 37177 |
| 2004 | 24168701 | 109985 | 18072 | 24204 | 35645 |
| 2005 | 24273381 | 110557 | 16816 | 24632 | 34815 |
| 2006 | 24380408 | 109531 | 16109 | 23657 | 33097 |
| 2007 | 24506227 | 110818 | 15809 | 24191 | 33399 |
| 2008 | 24647575 | 109181 | 14692 | 23634 | 32126 |
| 2009 | 24780338 | 109207 | 13844 | 24169 | 31767 |
| 2010 | 24902648 | 113047 | 13696 | 23647 | 31155 |
| 2011 | 25029688 | 114146 | 13006 | 22936 | 29875 |
| 2012 | 25157831 | 119849 | 12767 | 22531 | 29347 |
| 2013 | 25276024 | 119658 | 12505 | 22513 | 29017 |
| 2014 | 25388672 | 120371 | 12375 | 21758 | 28103 |
| 2015 | 25493662 | 125446 | 11933 | 21465 | 27615 |
| 2016 | 25585157 | 128298 | 11804 | 20826 | 26870 |
| 2017 | 25653952 | 131206 | 10969 | 19456 | 25047 |
| Total | 491609554 | 2291145 | 307630 | 458951 | 645257 |

Note: The annual number of population by age and sex can be found the following link: http://kosis.kr/statHtml/statHtml.do?orgId=101&tblId=DT_1B040M1&conn_path=I2

Supplementary Table S2. The list of causes of death considered amenable and preventable

| **Condition group and cause** | **ICD-10 codes** | **Age** | **Amenable** | **Preventable** |
| --- | --- | --- | --- | --- |
| **Infections** | | | | |
| Tuberculosis | A15-A19, B90 | 0-74 | • | • |
| Selected invasive bacterial and protozoal infections | A38-A41, A46, A48.1, B50-B54, G00, G03, J02, L03 | 0-74 | • |  |
| Hepatitis C | B17.1, B18.2 | 0-74 | • | • |
| HIV/AIDS | B20-B24 | All | • | • |
| **Neoplasms** | | | | |
| Malignant neoplasm of lip, oral cavity and pharynx | C00-C14 | 0-74 |  | • |
| Malignant neoplasm of oesophagus | C15 | 0-74 |  | • |
| Malignant neoplasm of stomach | C16 | 0-74 |  | • |
| Malignant neoplasm of colon and rectum | C18-C21 | 0-74 | • | • |
| Malignant neoplasm of liver | C22 | 0-74 |  | • |
| Malignant neoplasm of trachea, bronchus and lung | C33-C34 | 0-74 |  | • |
| Malignant melanoma of skin | C43 | 0-74 | • | • |
| Mesothelioma | C45 | 0-74 |  | • |
| Malignant neoplasm of breast | C50 | 0-74 | • | • |
| Malignant neoplasm of cervix uteri | C53 | 0-74 | • | • |
| Malignant neoplasm of bladder | C67 | 0-74 | • |  |
| Malignant neoplasm of thyroid gland | C73 | 0-74 | • |  |
| Hodgkin's disease | C81 | 0-74 | • |  |
| Leukaemia | C91, C92.0 | 0-44 | • |  |
| Benign neoplasms | D10-D36 | 0-74 | • |  |
| **Nutritional, endocrine and metabolic** | | | | |
| Diabetes mellitus | E10-E14 | 0-49 | • | • |
| **Drug use disorders** | | | | |
| Alcohol related diseases, excluding external causes | F10, G31.2, G62.1, I42.6, K29.2, K70, K73, K74 (excl. K74.3- K74.5), K86.0 | 0-74 |  | • |
| Illicit drug use disorders | F11-F16, F18-F19 | 0-74 |  | • |
| **Neurological disorders** | | | | |
| Epilepsy and status epilepticus | G40-G41 | 0-74 | • |  |
| **Cardiovascular diseases** | | | | |
| Rheumatic and other valvular heart disease | I01-I09 | 0-74 | • |  |
| Hypertensive diseases | I10-I15 | 0-74 | • |  |
| Ischaemic heart disease | I20-I25 | 0-74 | • | • |
| DVT with pulmonary embolism | I26, I80.1-I80.3, I80.9, I82.9 | 0-74 |  | • |
| Cerebrovascular diseases | I60-I69 | 0-74 | • |  |
| Aortic aneurysm and dissection | I71 | 0-74 |  | • |
| **Respiratory diseases** | | | | |
| Influenza (including swine flu) | J09-J11 | 0-74 | • | • |
| Pneumonia | J12-J18 | 0-74 | • |  |
| Chronic obstructive pulmonary disorder | J40-J44 | 0-74 |  | • |
| Asthma | J45-J46 | 0-74 | • |  |
| **Digestive disorders** | | | | |
| Gastric and duodenal ulcer | K25-K28 | 0-74 | • |  |
| Acute abdomen, appendicitis, intestinal obstruction, cholecystitis/lithiasis, pancreatitis, hernia | K35-K38, K40-K46, K80-K83, K85, K86.1- K86.9, K91.5 | 0-74 | • |  |
| **Genitourinary disorders** | | | | |
| Nephritis and nephrosis | N00-N07, N17-N19, N25-N27 | 0-74 | • |  |
| Obstructive uropathy and prostatic hyperplasia | N13, N20-N21, N35, N40, N99.1 | 0-74 | • |  |
| **Maternal and infant** | | | | |
| Complications of perinatal period | P00-P96, A33 | All | • |  |
| Congenital malformations, deformations and chromosomal anomalies | Q00-Q99 | 0-74 | • |  |
| **Unintentional injuries** | | | | |
| Transport Accidents | V01-V99 | All |  | • |
| Accidental Injury | W00-X59 | All |  | • |
| **Intentional injuries** | | | | |
| Suicide and self inflicted injuries | X60-X84, Y10-Y34 | All |  | • |
| Homicide/Assault | X85-Y09, U50.9 | All |  | • |
| Misadventures to patients during surgical and medical care | Y60-Y69, Y83-Y84 | All | • | • |

Adapted from Office for National Statistics (ONS), UK, 2011 (http://www.ons.gov.uk/ons/about-ons/get-involved/consultations/archived-consultations/2011/definitions-of-avoidable-mortality/definition-of-avoidable-mortality.pdf)

Supplementary Table S3. Life expectancy at birth and annual increases (years) from 1998 to 2017

| Year | Men and women combined | | Men | | Women | |
| --- | --- | --- | --- | --- | --- | --- |
|  | Life expectancy | Annual increases | Life expectancy | Annual increases | Life expectancy | Annual increases |
| 1998 | 75.40 | - | 71.39 | - | 79.20 | - |
| 1999 | 75.69 | 0.29 | 71.81 | 0.42 | 79.34 | 0.14 |
| 2000 | 75.96 | 0.27 | 72.21 | 0.40 | 79.48 | 0.14 |
| 2001 | 76.61 | 0.65 | 72.88 | 0.67 | 80.07 | 0.59 |
| 2002 | 76.93 | 0.32 | 73.32 | 0.44 | 80.26 | 0.19 |
| 2003 | 77.42 | 0.49 | 73.80 | 0.48 | 80.76 | 0.50 |
| 2004 | 77.94 | 0.52 | 74.30 | 0.50 | 81.26 | 0.50 |
| 2005 | 78.42 | 0.48 | 74.90 | 0.60 | 81.57 | 0.31 |
| 2006 | 78.95 | 0.53 | 75.41 | 0.51 | 82.08 | 0.51 |
| 2007 | 79.38 | 0.43 | 75.84 | 0.43 | 82.49 | 0.41 |
| 2008 | 80.02 | 0.64 | 76.36 | 0.52 | 83.19 | 0.70 |
| 2009 | 80.46 | 0.44 | 76.79 | 0.43 | 83.62 | 0.43 |
| 2010 | 80.65 | 0.19 | 76.96 | 0.17 | 83.81 | 0.19 |
| 2011 | 81.09 | 0.44 | 77.41 | 0.45 | 84.20 | 0.39 |
| 2012 | 81.28 | 0.19 | 77.66 | 0.25 | 84.37 | 0.17 |
| 2013 | 81.83 | 0.55 | 78.27 | 0.61 | 84.86 | 0.49 |
| 2014 | 82.21 | 0.38 | 78.71 | 0.44 | 85.19 | 0.33 |
| 2015 | 82.56 | 0.35 | 79.09 | 0.38 | 85.54 | 0.35 |
| 2016 | 82.78 | 0.22 | 79.39 | 0.30 | 85.73 | 0.19 |
| 2017 | 83.13 | 0.35 | 79.78 | 0.39 | 86.09 | 0.36 |
| Total |  | 7.73 |  | 8.39 |  | 6.89 |

Supplementary Table S4. Cause-specific contributions to the life expectancy difference (years) between 1998 and 2017

| **Causes** | **Amenable** | | | **Preventable** | | | **Avoidable** | | |
| --- | --- | --- | --- | --- | --- | --- | --- | --- | --- |
|  | All | Men | Women | All | Men | Women | All | Men | Women |
| **Infections** |  |  |  |  |  |  |  |  |  |
| Tuberculosis | 0.137 | 0.198 | 0.063 | 0.137 | 0.198 | 0.063 | 0.137 | 0.198 | 0.063 |
| Selected invasive bacterial and protozoal infections | 0.060 | 0.075 | 0.043 |  |  |  | 0.060 | 0.075 | 0.043 |
| Hepatitis C | -0.001 | -0.001 | -0.001 | -0.001 | -0.001 | -0.001 | -0.001 | -0.001 | -0.001 |
| HIV/AIDS | -0.002 | -0.003 | 0.000 | -0.002 | -0.003 | 0.000 | -0.002 | -0.003 | 0.000 |
| **Neoplasms** |  |  |  |  |  |  |  |  |  |
| Malignant neoplasm of lip, oral cavity and pharynx |  |  |  | -0.001 | 0.001 | 0.001 | -0.001 | 0.001 | 0.001 |
| Malignant neoplasm of oesophagus |  |  |  | 0.043 | 0.077 | 0.008 | 0.043 | 0.077 | 0.008 |
| Malignant neoplasm of stomach |  |  |  | 0.391 | 0.493 | 0.272 | 0.391 | 0.493 | 0.272 |
| Malignant neoplasm of colon and rectum | 0.011 | -0.002 | 0.036 | 0.011 | -0.002 | 0.036 | 0.011 | -0.002 | 0.036 |
| Malignant neoplasm of liver |  |  |  | 0.278 | 0.405 | 0.138 | 0.278 | 0.405 | 0.138 |
| Malignant neoplasm of trachea, bronchus and lung |  |  |  | 0.183 | 0.302 | 0.078 | 0.183 | 0.302 | 0.078 |
| Malignant melanoma of skin | -0.001 | 0.000 | -0.002 | -0.001 | 0.000 | -0.002 | -0.001 | 0.000 | -0.002 |
| Mesothelioma |  |  |  | -0.002 | -0.002 | 0.000 | -0.002 | -0.002 | 0.000 |
| Malignant neoplasm of breast | -0.019 | 0.000 | -0.048 | -0.019 | 0.000 | -0.048 | -0.019 | 0.000 | -0.048 |
| Malignant neoplasm of cervix uteri | 0.011 | 0.000 | 0.022 | 0.011 | 0.000 | 0.022 | 0.011 | 0.000 | 0.022 |
| Malignant neoplasm of bladder | 0.006 | 0.010 | 0.003 |  |  |  | 0.006 | 0.010 | 0.003 |
| Malignant neoplasm of thyroid gland | 0.004 | 0.003 | 0.005 |  |  |  | 0.004 | 0.003 | 0.005 |
| Hodgkin's disease | -0.001 | -0.001 | 0.000 |  |  |  | -0.001 | -0.001 | 0.000 |
| Leukaemia | 0.002 | 0.001 | 0.003 |  |  |  | 0.002 | 0.001 | 0.003 |
| Benign neoplasms | 0.001 | 0.001 | 0.001 |  |  |  | 0.001 | 0.001 | 0.001 |
| **Nutritional, endocrine and metabolic** |  |  |  |  |  |  |  |  |  |
| Diabetes mellitus | 0.032 | 0.045 | 0.015 | 0.032 | 0.045 | 0.015 | 0.032 | 0.045 | 0.015 |
| **Drug use disorders** |  |  |  |  |  |  |  |  |  |
| Alcohol related diseases, excluding external causes |  |  |  | 0.442 | 0.699 | 0.127 | 0.442 | 0.699 | 0.127 |
| Illicit drug use disorders |  |  |  | 0.000 | 0.000 | 0.000 | 0.000 | 0.000 | 0.000 |
| **Neurological disorders** |  |  |  |  |  |  |  |  |  |
| Epilepsy and status epilepticus | 0.013 | 0.014 | 0.011 |  |  |  | 0.013 | 0.014 | 0.011 |
| **Cardiovascular diseases** |  |  |  |  |  |  |  |  |  |
| Rheumatic and other valvular heart disease | 0.002 | 0.001 | 0.003 |  |  |  | 0.002 | 0.001 | 0.003 |
| Hypertensive diseases | 0.103 | 0.095 | 0.108 |  |  |  | 0.103 | 0.095 | 0.108 |
| Ischaemic heart disease | 0.146 | 0.164 | 0.134 | 0.146 | 0.164 | 0.134 | 0.146 | 0.164 | 0.134 |
| DVT with pulmonary embolism |  |  |  | -0.002 | 0.000 | -0.003 | -0.002 | 0.000 | -0.003 |
| Cerebrovascular diseases | 0.992 | 0.999 | 0.957 |  |  |  | 0.992 | 0.999 | 0.957 |
| Aortic aneurysm and dissection |  |  |  | 0.001 | -0.001 | 0.004 | 0.001 | -0.001 | 0.004 |
| **Respiratory diseases** |  |  |  |  |  |  |  |  |  |
| Influenza (including swine flu) | 0.001 | 0.001 | 0.001 | 0.001 | 0.001 | 0.001 | 0.001 | 0.001 | 0.001 |
| Pneumonia | 0.004 | 0.006 | 0.014 |  |  |  | 0.004 | 0.006 | 0.014 |
| Chronic obstructive pulmonary disorder |  |  |  | 0.037 | 0.047 | 0.031 | 0.037 | 0.047 | 0.031 |
| Asthma | 0.078 | 0.095 | 0.056 |  |  |  | 0.078 | 0.095 | 0.056 |
| **Digestive disorders** |  |  |  |  |  |  |  |  |  |
| Gastric and duodenal ulcer | 0.017 | 0.024 | 0.008 |  |  |  | 0.017 | 0.024 | 0.008 |
| Acute abdomen, appendicitis, intestinal obstruction, cholecystitis/lithiasis, pancreatitis, hernia | 0.018 | 0.024 | 0.011 |  |  |  | 0.018 | 0.024 | 0.011 |
| **Genitourinary disorders** |  |  |  |  |  |  |  |  |  |
| Nephritis and nephrosis | 0.052 | 0.057 | 0.048 |  |  |  | 0.052 | 0.057 | 0.048 |
| Obstructive uropathy and prostatic hyperplasia | 0.001 | 0.001 | 0.000 |  |  |  | 0.001 | 0.001 | 0.000 |
| **Maternal and infant** |  |  |  |  |  |  |  |  |  |
| Complications of perinatal period | -0.088 | -0.095 | -0.080 |  |  |  | -0.088 | -0.095 | -0.080 |
| Congenital malformations, deformations and chromosomal anomalies | 0.018 | 0.017 | 0.019 |  |  |  | 0.018 | 0.017 | 0.019 |
| **Unintentional injuries** |  |  |  |  |  |  |  |  |  |
| Transport Accidents |  |  |  | 0.598 | 0.806 | 0.339 | 0.598 | 0.806 | 0.339 |
| Accidental Injury |  |  |  | 0.385 | 0.527 | 0.216 | 0.385 | 0.527 | 0.216 |
| **Intentional injuries** |  |  |  |  |  |  |  |  |  |
| Suicide and self inflicted injuries |  |  |  | 0.014 | 0.061 | -0.021 | 0.014 | 0.061 | -0.021 |
| Homicide/Assault |  |  |  | 0.043 | 0.046 | 0.037 | 0.043 | 0.046 | 0.037 |
| Misadventures to patients during surgical and medical care | 0 | 0 | 0.001 | 0 | 0 | 0.001 | 0.000 | 0.000 | 0.001 |
| Sum | 1.597 | 1.729 | 1.431 | 2.725 | 3.863 | 1.448 | 4.010 | 5.191 | 2.659 |

Supplementary Table S5. Contributions to cumulative increase in life expectancy (years) based on the life expectancy at 1998 by sex

| Age group | Cumulative increase in life expectancy | Amenable causes | Preventable causes | Avoidable causes | Other non-avoidable causes |
| --- | --- | --- | --- | --- | --- |
| **Men and women combined** | |  |  |  |  |
| 1999 | 0.289 | -0.078 | 0.166 | 0.112 | 0.177 |
| 2000 | 0.559 | -0.082 | 0.270 | 0.227 | 0.332 |
| 2001 | 1.212 | -0.041 | 0.441 | 0.422 | 0.790 |
| 2002 | 1.531 | -0.005 | 0.437 | 0.486 | 1.045 |
| 2003 | 2.024 | 0.138 | 0.490 | 0.664 | 1.361 |
| 2004 | 2.539 | 0.295 | 0.689 | 0.997 | 1.542 |
| 2005 | 3.021 | 0.508 | 0.859 | 1.357 | 1.664 |
| 2006 | 3.549 | 0.614 | 1.117 | 1.706 | 1.843 |
| 2007 | 3.982 | 0.730 | 1.224 | 1.902 | 2.080 |
| 2008 | 4.626 | 0.917 | 1.398 | 2.201 | 2.425 |
| 2009 | 5.067 | 1.053 | 1.452 | 2.375 | 2.693 |
| 2010 | 5.254 | 1.091 | 1.601 | 2.537 | 2.717 |
| 2011 | 5.695 | 1.211 | 1.773 | 2.799 | 2.896 |
| 2012 | 5.878 | 1.253 | 1.937 | 2.991 | 2.887 |
| 2013 | 6.429 | 1.362 | 2.100 | 3.229 | 3.200 |
| 2014 | 6.816 | 1.401 | 2.271 | 3.428 | 3.388 |
| 2015 | 7.159 | 1.464 | 2.421 | 3.616 | 3.543 |
| 2016 | 7.382 | 1.503 | 2.556 | 3.772 | 3.610 |
| 2017 | 7.732 | 1.598 | 2.728 | 4.010 | 3.721 |
| **Men** |  |  |  |  |  |
| 1999 | 0.418 | -0.061 | 0.263 | 0.214 | 0.204 |
| 2000 | 0.817 | -0.040 | 0.416 | 0.399 | 0.417 |
| 2001 | 1.486 | -0.019 | 0.677 | 0.663 | 0.822 |
| 2002 | 1.932 | 0.065 | 0.745 | 0.834 | 1.098 |
| 2003 | 2.405 | 0.200 | 0.837 | 1.036 | 1.370 |
| 2004 | 2.904 | 0.382 | 1.080 | 1.432 | 1.472 |
| 2005 | 3.507 | 0.609 | 1.381 | 1.931 | 1.576 |
| 2006 | 4.020 | 0.692 | 1.704 | 2.323 | 1.696 |
| 2007 | 4.451 | 0.827 | 1.911 | 2.636 | 1.815 |
| 2008 | 4.967 | 1.010 | 2.130 | 2.966 | 2.002 |
| 2009 | 5.396 | 1.154 | 2.237 | 3.195 | 2.201 |
| 2010 | 5.568 | 1.177 | 2.398 | 3.355 | 2.212 |
| 2011 | 6.023 | 1.316 | 2.614 | 3.670 | 2.354 |
| 2012 | 6.272 | 1.353 | 2.818 | 3.906 | 2.366 |
| 2013 | 6.874 | 1.489 | 3.060 | 4.232 | 2.642 |
| 2014 | 7.318 | 1.534 | 3.282 | 4.484 | 2.834 |
| 2015 | 7.702 | 1.595 | 3.494 | 4.732 | 2.970 |
| 2016 | 7.994 | 1.641 | 3.666 | 4.926 | 3.068 |
| 2017 | 8.387 | 1.731 | 3.864 | 5.191 | 3.196 |
| **Women** |  |  |  |  |  |
| 1999 | 0.135 | -0.098 | 0.057 | -0.006 | 0.141 |
| 2000 | 0.275 | -0.125 | 0.111 | 0.039 | 0.236 |
| 2001 | 0.869 | -0.061 | 0.182 | 0.157 | 0.712 |
| 2002 | 1.058 | -0.076 | 0.131 | 0.135 | 0.923 |
| 2003 | 1.554 | 0.075 | 0.130 | 0.275 | 1.278 |
| 2004 | 2.056 | 0.196 | 0.289 | 0.539 | 1.517 |
| 2005 | 2.365 | 0.388 | 0.303 | 0.727 | 1.638 |
| 2006 | 2.870 | 0.520 | 0.490 | 1.029 | 1.841 |
| 2007 | 3.281 | 0.609 | 0.477 | 1.080 | 2.201 |
| 2008 | 3.986 | 0.788 | 0.590 | 1.326 | 2.660 |
| 2009 | 4.411 | 0.907 | 0.585 | 1.432 | 2.979 |
| 2010 | 4.603 | 0.961 | 0.720 | 1.596 | 3.007 |
| 2011 | 4.997 | 1.055 | 0.837 | 1.789 | 3.208 |
| 2012 | 5.162 | 1.109 | 0.957 | 1.937 | 3.224 |
| 2013 | 5.651 | 1.183 | 1.026 | 2.071 | 3.580 |
| 2014 | 5.988 | 1.218 | 1.134 | 2.205 | 3.783 |
| 2015 | 6.336 | 1.289 | 1.211 | 2.329 | 4.008 |
| 2016 | 6.528 | 1.324 | 1.302 | 2.444 | 4.084 |
| 2017 | 6.884 | 1.431 | 1.449 | 2.659 | 4.224 |

Supplementary Table S6. Age-specific contributions to the life expectancy difference (years) between 1998 and 2017 by sex

| Age group | Age-specific contributions | Amenable causes | Preventable causes | Avoidable causes | Other non-avoidable causes |
| --- | --- | --- | --- | --- | --- |
| **Men and women combined** | |  |  |  |  |
| 0 | -0.033 | -0.061 | 0.016 | -0.045 | 0.012 |
| 1-4 | 0.109 | 0.020 | 0.063 | 0.083 | 0.026 |
| 5-9 | 0.076 | 0.010 | 0.053 | 0.062 | 0.014 |
| 10-14 | 0.057 | 0.007 | 0.035 | 0.041 | 0.017 |
| 15-19 | 0.137 | 0.012 | 0.100 | 0.109 | 0.028 |
| 20-24 | 0.131 | 0.009 | 0.107 | 0.113 | 0.019 |
| 25-29 | 0.132 | 0.016 | 0.097 | 0.108 | 0.025 |
| 30-34 | 0.150 | 0.024 | 0.104 | 0.118 | 0.032 |
| 35-39 | 0.229 | 0.045 | 0.155 | 0.182 | 0.048 |
| 40-44 | 0.304 | 0.066 | 0.209 | 0.248 | 0.056 |
| 45-49 | 0.418 | 0.105 | 0.272 | 0.337 | 0.081 |
| 50-54 | 0.459 | 0.121 | 0.264 | 0.356 | 0.103 |
| 55-59 | 0.636 | 0.177 | 0.335 | 0.477 | 0.159 |
| 60-64 | 0.796 | 0.274 | 0.346 | 0.579 | 0.217 |
| 65-69 | 0.899 | 0.356 | 0.310 | 0.619 | 0.280 |
| 70-74 | 0.999 | 0.414 | 0.252 | 0.616 | 0.382 |
| 75-79 | 0.987 | 0.000 | 0.016 | 0.016 | 0.971 |
| 80-84 | 0.706 | 0.000 | 0.005 | 0.005 | 0.701 |
| 85+ | 0.538 | 0.000 | -0.010 | -0.010 | 0.548 |
| **Total** | **7.732** | **1.598** | **2.728** | **4.010** | **3.721** |
|  |  |  |  |  |  |
| **Men** |  |  |  |  |  |
| 0 | -0.046 | -0.067 | 0.017 | -0.051 | 0.006 |
| 1-4 | 0.111 | 0.020 | 0.062 | 0.081 | 0.030 |
| 5-9 | 0.089 | 0.012 | 0.062 | 0.073 | 0.016 |
| 10-14 | 0.065 | 0.005 | 0.040 | 0.045 | 0.020 |
| 15-19 | 0.173 | 0.013 | 0.133 | 0.141 | 0.032 |
| 20-24 | 0.176 | 0.008 | 0.152 | 0.158 | 0.018 |
| 25-29 | 0.178 | 0.017 | 0.141 | 0.151 | 0.027 |
| 30-34 | 0.213 | 0.031 | 0.157 | 0.175 | 0.038 |
| 35-39 | 0.324 | 0.058 | 0.231 | 0.265 | 0.059 |
| 40-44 | 0.442 | 0.090 | 0.323 | 0.372 | 0.069 |
| 45-49 | 0.587 | 0.135 | 0.406 | 0.485 | 0.102 |
| 50-54 | 0.608 | 0.143 | 0.384 | 0.489 | 0.119 |
| 55-59 | 0.824 | 0.203 | 0.477 | 0.634 | 0.190 |
| 60-64 | 0.992 | 0.304 | 0.491 | 0.741 | 0.252 |
| 65-69 | 1.056 | 0.371 | 0.452 | 0.760 | 0.296 |
| 70-74 | 1.019 | 0.386 | 0.328 | 0.664 | 0.355 |
| 75-79 | 0.825 | 0.000 | 0.012 | 0.012 | 0.813 |
| 80-84 | 0.493 | 0.000 | 0.003 | 0.003 | 0.489 |
| 85+ | 0.257 | 0.000 | -0.009 | -0.009 | 0.266 |
| **Total** | **8.387** | **1.731** | **3.864** | **5.191** | **3.196** |
|  |  |  |  |  |  |
| **Women** |  |  |  |  |  |
| 0 | -0.019 | -0.054 | 0.015 | -0.039 | 0.020 |
| 1-4 | 0.105 | 0.020 | 0.063 | 0.083 | 0.022 |
| 5-9 | 0.059 | 0.009 | 0.039 | 0.047 | 0.012 |
| 10-14 | 0.047 | 0.008 | 0.027 | 0.035 | 0.012 |
| 15-19 | 0.093 | 0.011 | 0.062 | 0.070 | 0.023 |
| 20-24 | 0.080 | 0.010 | 0.055 | 0.060 | 0.020 |
| 25-29 | 0.078 | 0.014 | 0.046 | 0.056 | 0.022 |
| 30-34 | 0.071 | 0.016 | 0.037 | 0.047 | 0.024 |
| 35-39 | 0.106 | 0.029 | 0.058 | 0.074 | 0.032 |
| 40-44 | 0.122 | 0.034 | 0.059 | 0.084 | 0.038 |
| 45-49 | 0.187 | 0.064 | 0.090 | 0.136 | 0.051 |
| 50-54 | 0.260 | 0.089 | 0.106 | 0.179 | 0.081 |
| 55-59 | 0.394 | 0.140 | 0.156 | 0.276 | 0.118 |
| 60-64 | 0.567 | 0.235 | 0.183 | 0.390 | 0.178 |
| 65-69 | 0.801 | 0.352 | 0.207 | 0.521 | 0.279 |
| 70-74 | 1.036 | 0.454 | 0.217 | 0.615 | 0.421 |
| 75-79 | 1.175 | 0.000 | 0.026 | 0.026 | 1.149 |
| 80-84 | 0.934 | 0.000 | 0.010 | 0.010 | 0.924 |
| 85+ | 0.787 | 0.000 | -0.010 | -0.010 | 0.797 |
| **Total** | **6.884** | **1.431** | **1.449** | **2.659** | **4.224** |

Supplementary Figure S1. The major amenable and preventable causes of death contributed to life expectancy difference between 1998 and 2017 by sex
